# RIS-Aided Physical Layer Security with Imperfect CSI: A Robust Model-Driven Deep Learning Approach

**DOI:** 10.3390/e28040457

**Published:** 2026-04-16

**Authors:** Ruikai Miao, Zhiqun Song, Yong Li, Xingjian Li, Lizhe Liu, Guoyuan Shao, Bin Wang

**Affiliations:** 1 The 54th Research Institute of China Electronics Technology Group Corporation, Shijiazhuang 050081, China; 202320000271@std.uestc.edu.cn (R.M.); szq_sjz@163.com (Z.S.); 18200276858@163.com (X.L.); lizhe_liucom@163.com (L.L.); bin_wcom@163.com (B.W.); 2 National Key Laboratory of Advanced Communication Networks, Shijiazhuang 050081, China; 3 North Automatic Control Technology Institute, Taiyuan 030006, China; shaoguoyuan1987@126.com

**Keywords:** reconfigurable intelligent surface (RIS), physical layer security, CSI, model-driven, deep unfold network

## Abstract

Reconfigurable intelligent surface (RIS) emerges as a promising paradigm and offers a new perspective for physical layer security. In practice, imperfect eavesdropper channel state information (CSI) represents a critical challenge for RIS-aided physical layer security design. To tackle this issue, this paper investigates RIS-aided physical layer security enhancement under imperfect eavesdropper CSI and formulates a robust weighted sum secrecy rate maximization problem. To efficiently solve this problem, a model-driven deep learning approach is proposed. We begin by introducing the gradient descent–ascent algorithm to solve the optimization problem. Then we unfold this algorithm into a gated recurrent unit (GRU)-aided deep unfold network with trainable parameters. The proposed GRU-aided deep unfold network leverages GRU to adaptively generate gradient ascent–descent step sizes. Different from the existing deep unfold network that commonly has a fixed number of iteration, the proposed deep unfold network integrates the sequential learning capability of GRU and enables adaptive iteration adjustment. The simulation results demonstrate that compared to existing non-robust optimization algorithm and traditional deep unfold network with fixed number of iteration, the proposed method exhibits robustness against imperfect CSI and achieves higher weighted sum secrecy rate.

## 1. Introduction

### 1.1. Background

To meet the vision of intelligent connectivity and global coverage in the 6G era, wireless communication networks are evolving towards a more intelligent, integrated, and open direction [1]. However, while achieving ubiquitous connectivity, the inherent broadcast and open-access nature of the wireless channel bring security threats such as eavesdropping. Traditional security mechanisms mainly rely on encryption at the upper layer of the protocol stack [2]. This type of strategy is based on computational complexity and not only comes with high secret key management overhead, but also increasingly faces the threat of being attacked due to the fast development of advanced technologies such as quantum computing. In contrast, physical layer security exploits the randomness and time-varying nature of wireless channels as usable resources, directly safeguarding the transmission of confidential information in the physical layer [3]. Physical layer security offers the potential for information-theoretic security and does not require key distribution. Therefore, it is a promising approach to enhance the security of future wireless communications.

Reconfigurable intelligent surface (RIS) is emerging as a key technology for the dynamic and intelligent control of wireless environments [4]. RIS consists of a large number of programmable electromagnetic elements. These elements can adjust the amplitude and phase of incident wireless signals via software control, thereby intelligently reshaping the electromagnetic propagation environment. This promising technology has been widely investigated in several potential scenarios, such as coverage enhancement [5], integrated sensing and communications (ISAC) [6,7] and simultaneous wireless information and power transfer (SWIPT) [8]. RIS can establish communication links even when the direct link between transceivers is blocked. In such cases, traditional communication and physical layer security methods will fail to provide secure communication links. Moreover, traditional physical layer security methods primarily adapt to wireless channels. In contrast, RIS provides a novel approach that can adaptively reconstruct the wireless channels by steering the reflected signal to a desirable receiver while suppressing the information leakage to unauthorized receiver. RIS can provide new dimensions for the implementation of physical layer security [9] and has gained widespread attention from researchers.

### 1.2. Related Works

Motivated by these advantages, RIS-aided secure communication has been widely studied in the literature. Existing work on RIS-aided secure communication has considered both perfect and imperfect CSI. In the initial literature [10,11,12,13], the authors considered a single user multiple-input single-output (MISO) system in the presence of a single eavesdropper with perfect CSI. To maximize the secrecy rate, different alternating optimization (AO) algorithms were developed to efficiently optimize the beamforming vector of the base station and the RIS phase shift matrix. In [14], the authors investigated the multi-user MISO (MU-MISO) scenario and formulated a weighted sum secrecy rate maximization problem and developed an AO algorithm to address the optimization problem. In [15], RIS location optimization was also investigated to provide higher secrecy performance.

The above studies have revealed the potential application of RIS in physical layer security and reveal the performance bound of the proposed scheme with the assumption of perfect CSI. However, in practical scenarios, it is necessary to consider imperfect CSI of the eavesdropper. The reason lies in the following aspects. On one hand, eavesdroppers are usually covert and silent. Although auxiliary sensing methods can obtain approximate information about the eavesdropper, their accuracy is often limited, leading to imperfect CSI. On the other hand, the actual environment may be highly dynamic, and the obtained CSI may be outdated [16]. Furthermore, RIS lacks signal processing units, and channel estimation for RIS-aided channel is relatively challenging and may involve errors. Imperfect CSI is typically represented by a bounded CSI error model or statistical CSI error model. When imperfect CSI is considered, the joint beamforming problem is formulated as a robust optimization problem with highly coupled variables and CSI uncertainty constraints. Compared to the optimization problem under perfect CSI, the imperfect CSI poses further challenges to the joint beamforming design of RIS. In recent years, there have been several studies aimed at tackling the robust beamforming in RIS-aided physical layer security. The authors of [17] derived the eavesdropper’s CSI error bound related the accuracy of sensing methods, and subsequently formulated a worst-case robust optimization problem. Then, the robust beamforming method based on the S-procedure and the general sign definiteness condition was proposed to maximize the sum secrecy rate. In [18], the authors maximized the sum rate of the legitimate users under the constraint that the maximum information leakage to the eavesdropper was not higher than a threshold. Different from the aforementioned works which adopted bounded CSI error model, several works also adopt statistical CSI error model. In such case, the optimization problem is formulated as the maximization of secrecy rate under the constraint that the secrecy outage probability is lower than a threshold. To address this outage constraint robust optimization problem, the Berstein-type inequality is commonly used to transform the outage probability constraint into a safe approximation form [19,20].

In recent years, artificial intelligence algorithms have provided novel solutions to many challenges in physical layer communication [21,22,23,24,25]. In [16,26], deep reinforcement learning has been leveraged to provide beamforming solutions in dynamic environments. The data-driven deep learning method views the original problem as a black box. The neural network is leveraged to directly learn the non-linear mapping of the channel realizations and the beamforming parameters [27]. Among the deep learning methods, the deep unfold network, as a model-driven approach that integrates original model and neural network, has shown significant potential [28]. The core of the deep unfold network is to unfold each iteration of traditional optimization algorithms to a layer of the neural network, thereby constructing an interpretable and trainable end-to-end architecture. The key parameters in the traditional algorithm are converted into trainable parameters in the neural network. This type of method has received widespread attention and has been successfully applied in beamforming design [23,29,30,31]. To tackle the robust beamforming design, the authors of [32] proposed a liquid neural network (LNN)-aided method. This method unfolded the original gradient-based optimization structure and leveraged the ability of the LNN to extract features from imperfect CSI. The simulations verified its robustness against CSI imperfection. The authors in [33] utilized a deep unfold network to address the robust beamforming problem in a massive multiple-input multiple-output (MIMO) system. This method was based on the gradient descent/ascent algorithm and the step sizes of gradient descent/ascent were unfolded into trainable parameters of the network. In [34] an RIS-aided ISAC system was investigated, where the statistical location uncertainty of the target was considered. The robust beamforming design was formulated as a worst-case max–min optimization problem, a deep unfold network was proposed to address the robust beamforming. The challenge of such a deep unfold method is that the hyperparameters of the network (such as the number of layers) have a significant impact on performance, and these hyperparameters are usually adjusted empirically. In practical deployment scenarios, the location of RIS may change due to network topology reconfiguration. Meanwhile, the locations of users and potential eavesdroppers can also change. Such dynamics lead to fluctuations of channel characteristics. These factors will lead to the situation the required computational effort to guarantee secrecy performance can vary. These characteristics bring challenges to deep unfold network design.

### 1.3. Our Contributions

Motivated by the aforementioned works, we consider an RIS-aided MU-MISO secure communication system with imperfect CSI of the eavesdropper. A robust model-driven deep learning approach is proposed to address the robust and secure beamforming design. The key contributions are summarized as follows:

- We investigate an RIS-aided MU-MISO secure communication system with imperfect eavesdropper CSI. A robust weighted sum secrecy rate maximization problem is formulated.
- We propose a novel GRU-aided deep unfold network to address the robust weighted sum secrecy rate maximization problem. Different from the existing deep unfold network that commonly has fixed number of iteration, the proposed method enables adaptive iteration adjustment.
- The simulations show that compared to existing non-robust optimization algorithms and the traditional deep unfold network with fixed number of iteration, the proposed method shows robustness against CSI imperfection and achieves a higher weighted sum secrecy rate.

## 2. System Model and Problem Formulation

**Figure 1:**
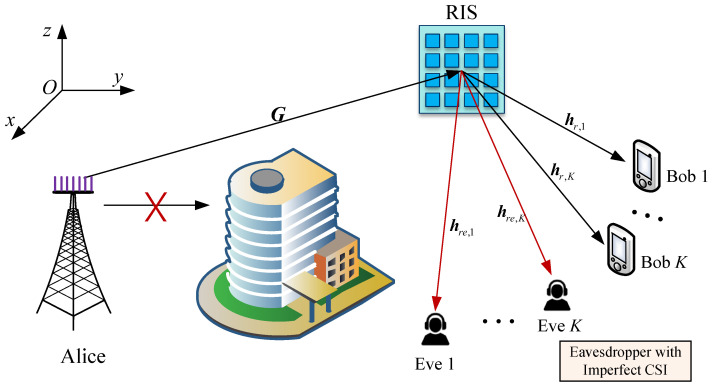
An RIS-assisted secure communication system eavesdroppers’ imperfect CSI.

This paper investigates an RIS-assisted narrow band downlink MU-MISO secure communication system, as illustrated in Figure 1. In this system, the direct links are blocked due to an obstacle. To overcome the blockage and establish high-quality reflection links, RIS is deployed near the legitimate users. Specifically, the system comprises a base station (Alice) equipped with an *M*-antenna uniform linear array (ULA), an RIS consisting of $N=N_{1}\times N_{2}$ passive reflecting elements in a uniform planar array (UPA), with $N_{1}$ elements horizontally and $N_{2}$ vertically. The antenna spacing of the ULA and UPA are both λ/2, where λ denotes the carrier wavelength. Alice transmits confidential signals to *K* single-antenna legitimate users (Bob) with the assistance of RIS.

The system operates in the far-field region, and the geometric locations of all nodes are defined within a right-handed 3D Cartesian coordinate system. We assume Alice is located at the origin of the coordinate system and the ULA of Alice is parallel to *x*-axis. The coordinates of RIS is denoted by $r={\left[r_{x},r_{y},r_{z}\right]}^{T}$ and UPA of RIS is parallel to *x*-*o*-*z* plane. The coordinate of the *k*-th Bob is $p_{k}={\left[p_{x,k},p_{y,k},p_{z,k}\right]}^{T}$.

### 2.1. Channel Model and Channel Error Model

Without the loss of generality, all channels are modeled as Rician fading. $G\in C^{N\times M}$ denotes the channel between Alice and RIS; it can be written as

(1) $$G=\sqrt{L_{AR}}\left(\sqrt{\frac{\kappa }{\kappa +1}}G^{\mathrm{LoS}}+\sqrt{\frac{1}{\kappa +1}}G^{\mathrm{NLoS}}\right),$$

where κ is the Rician factor. $L_{AR}$ is the free space path loss from Alice to RIS, which is given by

(2) $$L_{AR}=C_{0}{\left(\frac{d_{AR}}{D_{0}}\right)}^{-\alpha },$$

where $C_{0}$ is the path loss at the reference distance $D_{0}=1\;\left(m\right)$, $d_{AR}$ is the distance between Alice and RIS, and α is the path loss factor. $G^{\mathrm{LoS}}$ are the LoS components, and $G^{\mathrm{NLoS}}$ are the NLoS components, whose elements are chosen from CN (0,1). $G^{\mathrm{LoS}}$ is denoted by $G^{\mathrm{LoS}}=\alpha_{A}\left(\phi^{A,azi},\phi^{A,ele}\right)\cdot \alpha_{R}^{H}\left(\phi^{D,azi},\phi^{D,ele}\right)$, where $\alpha_{A}\left(\cdot \right)$ is the array response vector of Alice, $\alpha_{R}\left(\cdot \right)$ is the array response vector of RIS. $\phi^{D,azi}$ and $\phi^{D,ele}$ represent the azimuth angle-of-departure (AOD) and elevation AOD at Alice, and $\phi^{A,azi}$ and $\phi^{A,ele}$ represent the azimuth angle of arrival (AOA) and elevation AOA at RIS.

$h_{r,k}$ denotes the channel between RIS and *k*-th Bob; it can be written as

(3) $$h_{r,k}=\sqrt{L_{r,k}}\left(\sqrt{\frac{\kappa }{\kappa +1}}h_{r,k}^{\mathrm{LoS}}+\sqrt{\frac{1}{\kappa +1}}h_{r,k}^{\mathrm{NLoS}}\right),$$

where $L_{r,k}$ is the path loss related to the distance between RIS and the *k*-th Bob. The LoS component $h_{r,k}^{\mathrm{LoS}}$ is denoted by reflecting array response; it is given by $h_{r,k}^{\mathrm{LoS}}=\alpha_{R}\left(\phi_{k}^{D,azi},\phi_{k}^{D,ele}\right)$, where $\phi_{k}^{D,azi}$ and $\phi_{k}^{D,ele}$ represent the azimuth AOD and elevation AOD at RIS.

The CSI of legitimate channels G and $h_{r,k}$ can be obtained by channel estimation techniques such as [35,36]. $h_{re,k}\in C^{N\times 1}$ denotes the channel between RIS and *k*-th Eve. The assumption of perfect CSI is commonly adopted to reveal the performance bound. However, in practical scenarios, the eavesdropper is a non-cooperative and silent node that does not transmit any pilot or training signals. Consequently, Alice has no direct means to estimate the channel to the eavesdropper through conventional channel estimation techniques. Although advanced methods like sensing local oscillator leakage [37] or exploiting environment sensing techniques such as ISAC [17,38] have been proposed, they can only provide coarse and imperfect channel information. Therefore, to capture the practical challenge where only imperfect or partial knowledge of the eavesdropper’s channel is available, imperfect CSI must be considered. In this work, we adopt a bounded error model based on worst-case optimization paradigm. The CSI error bound can be set based on the accuracy and resolution of the sensing technology employed. Therefore the imperfect channel $h_{re,k}$ can be expressed as follows:

(4) $$h_{re,k}={\hat{h}}_{re,k}+\Delta h_{re,k},$$

where $∥\Delta h_{re,k}∥$ satisfies $∥\Delta h_{re,k}∥\;\leq \rho_{k}$.

### 2.2. Signal Transmission Model and Problem Formulation

The transmit signal from Alice can be denoted as

(5) $$x=\sum_{k=1}^{K}w_{k}s_{k},$$

where $w_{k}\in C^{M\times 1}$ denotes the corresponding beamforming vector intent to the *k*-th Bob, and is the *k*-th column of precoder matrix $W\in C^{M\times K}$. $s_{k}$ is the normalized confidential message to the *k*-th Bob with zero mean and unit variance. The phase shift matrix of RIS is defined as $\Theta =\mathrm{diag}\left(\theta \right)=\mathrm{diag}\left(\theta_{1},\theta_{2},\ldots ,\theta_{N}\right)=\mathrm{diag}\left(e^{j\varphi_{1}},e^{j\varphi_{2}},\ldots ,e^{j\varphi_{N}}\right)$. Thus, the received signals at the *k*-th Bob and the *k*-th Eve can be given by

(6) $$\begin{array}{cc} \; & y_{b,k}=h_{r,k}^{H}\Theta G\sum_{k=1}^{K}w_{k}s_{k}+n_{b,k}, \end{array}$$

(7) $$\begin{array}{cc} \; & y_{e,k}=h_{re,k}^{H}\Theta G\sum_{k=1}^{K}w_{k}s_{k}+n_{e,k}, \end{array}$$

where $n_{b,k}$ and $n_{e,k}$ is the additive white Gaussian noise at receiver with power $\sigma_{b,k}^{2}$ and $\sigma_{e,k}^{2}$. The signal-to-interference-plus-noise ratio (SINR) of the *k*-th Bob/Eve is expressed as

(8) $$\begin{array}{cc} \; & \gamma_{b,k}=\frac{{\left|h_{r,k}^{H}\Theta Gw_{k}\right|}^{2}}{\sum_{i=1,i\neq k}^{K}{\left|h_{r,k}^{H}\Theta Gw_{i}\right|}^{2}+\sigma_{b,k}^{2}}, \end{array}$$

(9) $$\begin{array}{cc} \; & \gamma_{e,k}=\frac{{\left|h_{re,k}^{H}\Theta Gw_{k}\right|}^{2}}{\sum_{i=1,i\neq k}^{K}{\left|h_{re,k}^{H}\Theta Gw_{i}\right|}^{2}+\sigma_{e,k}^{2}}, \end{array}$$

In this paper, we aim to maximize the worst-case weighted sum secrecy rate by joint active/passive beamforming. The optimization problem can be formulated as

(10a) $$\begin{array}{cc} \left(P1\right):\max_{w_{k},\Theta } & \min_{\begin{array}{c} \Delta h_{re,k} \end{array}}R_{S}=\sum_{k=1}^{K}\psi_{k}{\left[R_{b,k}-R_{e,k}\right]}^{+} \end{array}$$

(10b) $$\begin{array}{cc} s.t.\; & \sum_{k=1}^{K}{∥w_{k}∥}^{2}\leq P, \end{array}$$

(10c) $$\begin{array}{cc} \; & \left|e^{j\varphi_{n}}\right|=1,\;1\leq n\leq N, \end{array}$$

(10d) $$\begin{array}{cc} \; & ∥\Delta h_{re,k}∥\;\leq \rho_{k},\;1\leq k\leq K, \end{array}$$

where $R_{b,k}=\log_{2}\left(1+\gamma_{b,k}\right)$ and $R_{e,k}=\log_{2}\left(1+\gamma_{e,k}\right)$, respectively. $\psi_{k}$ is the weighted coefficient for the *k*-th user. $\rho_{k}$ is the CSI error bound of $h_{re,k}$ by utilizing norm-bounded error model. The operator ${\left[z\right]}^{+}=\max\left(z,0\right)$ can be omitted without the loss of optimality, since the maximum sum secrecy rate must be non-negative. (P1) is non-convex because of several challenges. (1) The precoder matrix W and phase shift matrix Θ are coupled in the objective function; (2) Each RIS phase shift $e^{j\varphi_{n}}$ must satisfy constant-modulus in (10c); (3) The CSI uncertainty in (10d) is hard to deal with. To address this intractable problem, we will propose an optimization framework in the next section.

## 3. Proposed Model-Driven Deep Learning Approach

In this section, we propose the model-driven deep learning approach to address the robust and secure beamforming problem. We begin by introducing the gradient descent–ascent algorithm. We then unfold this algorithm into a GRU-aided deep unfold network with trainable parameters. The proposed method introduces flexible iteration adjustment, which allows the network to adaptively determine the number of iterations based on convergence condition.

### 3.1. Gradient Descent–Ascent Algorithm

The gradient descent–ascent algorithm is an effective approach for addressing the max–min problem [33,34,39]. The inner minimization captures the worst-case channel uncertainty, while the outer maximization optimizes system performance under the given worst-case channel conditions. Specifically, in robust secure beamforming problem (P1), the inner minimization subproblem treats the CSI uncertainty as an auxiliary variable to obtain the worst-case CSI. Subsequently, the outer maximization subproblem employs a alternating gradient ascent method to alternately optimize the precoder matrix and the RIS phase shift, thereby maximizing the weighted sum secrecy rate. The detailed procedure is given as follows.

During the initialization phase, the optimization variables are initialized as follows. the RIS phase shift Θ is initialized randomly. The channel uncertainty $\Delta h_{re,k}$ is treated as an auxiliary variable; it is initialized while satisfying the uncertainty bound. The beamforming vector $w_{k}$ is initialized as $w_{k}={\left(h_{r,k}^{H}\Theta G\right)}^{H}$. Thus, the precoder matrix W can be initialized corresponding to the legitimate channel, which provides a feasible starting point for the optimization algorithm. It can be given as follows:

(11) $$W^{\left(0\right)}=\left[w_{1},w_{2},\ldots ,w_{K}\right]\sqrt{\frac{P}{\sum_{k=1}^{K}{∥w_{k}∥}^{2}}}.$$

We first address the inner minimization subproblem using the projected gradient descent (PGD) method, where the update of $\Delta h_{re,k}$ can be expressed by

(12) $$\Delta {\hat{h}}_{re,k}^{\left(t\right)}=\Delta h_{re,k}^{\left(t-1\right)}-\mu_{1,k}^{\left(t\right)}\nabla_{\Delta h_{re,k}}R_{S},$$

where $\mu_{1,k}^{\left(t\right)}$ denotes the step size in *t*-iteration for updating $\Delta h_{re,k}$. To derive $\nabla_{\Delta h_{re,k}}R_{S}$, $R_{e,k}$ can be first rewritten as

(13) $$\begin{array}{cc} R_{e,k} & =\log_{2}\left(\frac{\sum_{i=1}^{K}{\left|\left({\hat{h}}_{re,k}+\Delta h_{re,k}\right)\Theta Gw_{i}\right|}^{2}}{\sum_{i=1,i\neq k}^{K}{\left|\left({\hat{h}}_{re,k}+\Delta h_{re,k}\right)\Theta Gw_{i}\right|}^{2}+\sigma_{e,k}^{2}}\right)\\ \; & =\log_{2}\left(\sum_{i=1}^{K}{\left|\left({\hat{h}}_{re,k}+\Delta h_{re,k}\right)\Theta Gw_{i}\right|}^{2}\right)-\log_{2}\left(\sum_{i=1,i\neq k}^{K}{\left|\left({\hat{h}}_{re,k}+\Delta h_{re,k}\right)\Theta Gw_{i}\right|}^{2}+\sigma_{e,k}^{2}\right). \end{array}$$

Thus the gradient $\nabla_{\Delta h_{re,k}}R_{S}=-\psi_{k}\nabla_{\Delta h_{re,k}}R_{e,k}$ can be expressed as follows:

(14) $$\begin{array}{cc} \nabla_{\Delta h_{re,k}}R_{S}= & -\psi_{k}[\frac{\sum_{i=1}^{K}\Theta Gw_{i}{\left(\Theta Gw_{i}\right)}^{H}\left({\hat{h}}_{re,k}+\Delta h_{re,k}\right)}{\sum_{i=1}^{K}{\left|\left({\hat{h}}_{re,k}+\Delta h_{re,k}\right)\Theta Gw_{i}\right|}^{2}}\\ & -\frac{\sum_{i=1,i\neq k}^{K}\Theta Gw_{i}{\left(\Theta Gw_{i}\right)}^{H}\left({\hat{h}}_{re,k}+\Delta h_{re,k}\right)+\sigma_{e,k}^{2}}{\sum_{i=1,i\neq k}^{K}{\left|\left({\hat{h}}_{re,k}+\Delta h_{re,k}\right)\Theta Gw_{i}\right|}^{2}+\sigma_{e,k}^{2}}], \end{array}$$

Then, the projection operation is adopted to project $\Delta {\hat{h}}_{re,k}^{\left(t\right)}$ to the channel uncertainty bound. It can be given as

(15) $$\Delta h_{re,k}^{\left(t\right)}=\left\{\begin{array}{cc} \Delta {\hat{h}}_{re,k}^{\left(t\right)} & ∥\Delta {\hat{h}}_{re,k}^{\left(t\right)}∥\leq \rho_{k}\\ \frac{\rho_{k}}{∥\Delta {\hat{h}}_{re,k}^{\left(t\right)}∥}\Delta {\hat{h}}_{re,k}^{\left(t\right)} & \mathrm{otherwise}. \end{array}\right.$$

Then, we solve the outer maximization subproblem under the worst-case CSI in *t*-th iteration $h_{re,k}={\hat{h}}_{re,k}+\Delta h_{re,k}^{\left(t\right)}$. Since the optimization variables are coupled, we optimize W and θ alternatively. The update of the precoder matrix W is implemented via project gradient ascent (PGA) method. It can be expressed by

(16) $${\hat{W}}^{\left(t\right)}=W^{\left(t-1\right)}+\mu_{2}^{\left(t\right)}\nabla_{W}R_{S},$$

where $\mu_{2}^{\left(t\right)}$ denotes the step size in the *t*-iteration for updating W. The expression of $\nabla_{W}R_{S}$ can be obtained as follows. First, $\nabla_{W}R_{S}$ can be written as

(17) $$\nabla_{W}R_{S}=\sum_{i=1}^{K}\psi_{k}\left(R_{b,k}-R_{e,k}\right).$$

The derivation of $\nabla_{W}R_{S}$ can be obtained through the following steps. According to [40], $\nabla_{W}R_{b,k}$ can be expressed as $\nabla_{W}R_{b,k}=\left[\frac{\partial R_{b,k}}{\partial w_{1},},\frac{\partial R_{b,k}}{\partial w_{2},},\ldots ,\frac{\partial R_{b,k}}{\partial w_{K},}\right]$. To simplify the expression, we denote $h_{k}=h_{r,k}^{H}\Theta G$; $R_{b,k}$ can be rewritten as

(18) $$\begin{array}{cc} R_{b,k}= & \log_{2}\left(\frac{\sum_{i=1}^{K}{\left|h_{k}w_{i}\right|}^{2}+\sigma_{b,k}^{2}}{\sum_{i=1,i\neq k}^{K}{\left|h_{k}w_{i}\right|}^{2}+\sigma_{b,k}^{2}}\right)\\ = & \log_{2}\left(\sum_{i=1}^{K}{\left|h_{k}w_{i}\right|}^{2}+\sigma_{b,k}^{2}\right)-\log_{2}\left(\sum_{i=1,i\neq k}^{K}{\left|h_{k}w_{i}\right|}^{2}+\sigma_{b,k}^{2}\right). \end{array}$$

Therefore, $\nabla_{w_{k}}R_{b,k}$ can be expressed as

(19) $$R_{b,k}=\frac{2}{\ln2}\frac{h_{k}h_{k}^{H}w_{k}}{\sum_{i=1}^{K}{\left|h_{k}w_{i}\right|}^{2}+\sigma_{b,k}^{2}}.$$

$\nabla_{w_{k}}R_{b,i}$ can be expressed as

(20) $$\nabla_{w_{k}}R_{b,i}=\frac{2}{\ln2}\left(\frac{h_{k}w_{k}}{\sum_{i=1}^{K}{\left|h_{k}w_{i}\right|}^{2}+\sigma_{b,k}^{2}}-\frac{h_{k}w_{k}}{\sum_{i=1,i\neq k}^{K}{\left|h_{k}w_{i}\right|}^{2}+\sigma_{b,k}^{2}}\right)h_{k}^{H}.$$

$\nabla_{W}R_{e,k}$ can be similarly obtained according to (19) and (20). By combining the gradient $\nabla_{W}R_{e,k}$ and $\nabla_{W}R_{b,k}$, (14) can be obtained. Then, projection is performed to satisfy the total power constraint

(21) $$W^{\left(t\right)}=\left\{\begin{array}{cc} {\hat{W}}^{\left(t\right)} & \mathrm{Tr}({\hat{W}}^{\left(t\right)}{\left({\hat{W}}^{\left(t\right)}\right)}^{H})\leq P\\ \frac{\sqrt{P}}{∥{\hat{W}}^{\left(t\right)}∥}{\hat{W}}^{\left(t\right)} & \mathrm{otherwise}. \end{array}\right.$$

To optimize the RIS phase shift θ, we employ Riemannian manifold optimization (RMO). Specifically, the subproblem of updating the phase shifts can be viewed as an unconstrained optimization on an *N*-dimension Riemannian complex circle manifold, defined as $\mathrm{CCM}=\{\theta \in C^{N\times 1}:\left|\theta_{1}\right|=\left|\theta_{2}\right|=\cdot \cdot \cdot =\left|\theta_{N}\right|=1\}$. The update of phase shift can be derived within this manifold framework.

(22) $${\tilde{\theta }}^{\left(t\right)}=\theta^{\left(t-1\right)}+\mu_{3}^{\left(t\right)}{\mathrm{grad}}_{\theta }R_{S},$$

where ${\mathrm{grad}}_{\theta }R_{S}$ denotes the Riemannian gradient on CCM, which represents the steepest ascent direction of $R_{S}$ under the unit modulus constraint. The Riemannian gradient is obtained by orthogonally projecting the standard Euclidean gradient onto the tangent space of the manifold. ${\mathrm{grad}}_{\theta }R_{S}$ can be calculated as

(23) gradθRS=∇θRS−Re{∇θRS∘(θ(t−1))*}∘θ(t−1).

In (23), the key variables and operators are defined as follows:

- $\nabla R_{S}$ is the Euclidean gradient of $R_{S}$ with respect to θ, whose explicit expression is given in (24) below.
- $\theta^{\left(t-1\right)}$ is the phase shift from the previous iteration.
- The operator ${\left(\cdot \right)}^{*}$ denotes the complex conjugate.
- The operator Rex takes the real part of each element of x.
- ∘ is the Hadamard product.

$\nabla_{\theta }R_{S}$ is derived as

(24) $$\begin{array}{cc} \nabla_{\theta }R_{S}= & 2\psi_{k}\sum_{k=1}^{K}[\left(\frac{\sum_{i=1}^{K}a_{i,k,1}a_{i,k,1}^{H}\theta }{\sum_{i=1}^{K}{\left|\theta^{H}a_{i,k,1}\right|}^{2}+\sigma_{b,k}^{2}}-\frac{\sum_{i\neq k}^{K}a_{i,k,1}a_{i,k,1}^{H}\theta }{\sum_{i\neq k}^{K}{\left|\theta^{H}a_{i,k,1}\right|}^{2}+\sigma_{b,k}^{2}}\right)\\ & -\left(\frac{\sum_{i=1}^{K}a_{i,k,2}a_{i,k,2}^{H}\theta }{\sum_{i=1}^{K}{\left|\theta^{H}a_{i,k,2}\right|}^{2}+\sigma_{e,k}^{2}}-\frac{\sum_{i\neq k}^{K}a_{i,k,2}a_{i,k2}^{H}\theta }{\sum_{i\neq k}^{K}{\left|\theta^{H}a_{i,k,2}\right|}^{2}+\sigma_{e,k}^{2}}\right)], \end{array}$$

where

(25) $$\begin{array}{cc} a_{i,k,1} & =\mathrm{diag}\left(h_{r,k}^{H}G\right)w_{i}, \end{array}$$

(26) $$\begin{array}{cc} a_{i,k,2} & =\mathrm{diag}\left(h_{re,k}^{H}G\right)w_{i}. \end{array}$$

Finally, retraction operation is performed to project ${\tilde{\theta }}^{\left(t\right)}$ onto the complex circle manifold

(27) $$\theta^{\left(t\right)}=\frac{{\tilde{\theta }}^{\left(t\right)}}{\left|{\tilde{\theta }}^{\left(t\right)}\right|}.$$

### 3.2. GRU-Aided Deep Unfold Network

The step size plays an important role in the performance of optimization algorithms. To obtain better step sizes, the traditional algorithm can be unfolded into trainable layers and combines the learning capabilities of the neural network to achieve better step sizes. However, existing deep unfold networks employ a fixed number of iterations and these usually determined empirically. In practice, the potential change of the users or eavesdroppers’ locations will lead to fluctuations in the channel characteristics. The fixed number of iterations will lead to a lack of flexibility for different channel realizations. That means that the deep unfold network may use insufficient iterations for certain channel realizations, which require more iterations to achieve satisfactory performance, or, conversely, employ redundant iterations for other channel realizations.

**Figure 2:**
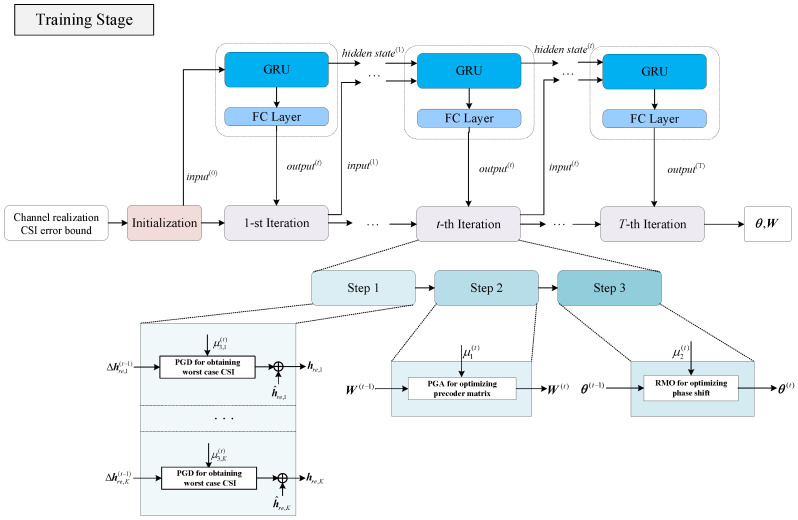
Training stage of the proposed GRU-aided deep unfold network.

**Figure 3:**
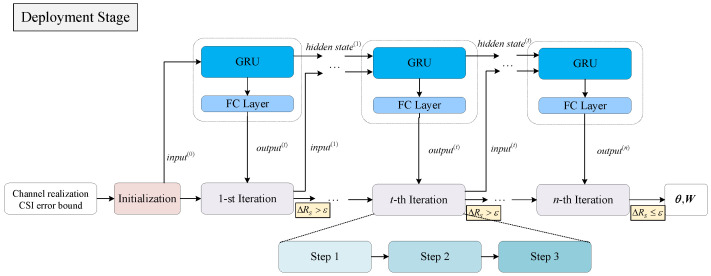
Deployment stage of the GRU-aided deep unfold network with adaptive iteration adjustment.

In this subsection, we propose the GRU-aided deep unfold network. This method unfolds the iteration of the gradient descent–ascent algorithm, where the step sizes of gradient descent–ascent are trainable variables. The generation of step sizes can be viewed as a sequence decision task. There exist potential non-linear patterns between the step sizes in different iterations, and GRU is inherently able to capture such temporal dependencies and complex patterns in sequential data. Therefore we integrate the sequential processing capability of GRU with the model-based iterative algorithm framework. To be specific, building on the aforementioned gradient descent–ascent algorithm, the proposed deep unfold network leverages GRU to adaptively generate gradient ascent–descent step sizes. This design enables the network to dynamically generate the step size for each iteration based on real-time channel conditions and convergence status. Furthermore, it determines when to terminate the iterative process according to predefined convergence conditions. Consequently, the network depth is adapted. The workflows of the proposed method in the training and deployment stages are shown in Figure 2 and Figure 3.

The value of the step sizes is highly related to the convergence process. Larger step sizes may be preferred during the initial iterations, and the smaller step sizes may be preferred as the algorithm nears convergence. In *t*-iteration, the input set of GRU is defined as

(28) $$input^{\left(t\right)}=\left[t,R_{S}^{\left(t-1\right)},\Delta R_{S},\mu_{1}^{\left(t-1\right)},\mu_{2}^{\left(t-1\right)},\mu_{3,1}^{\left(t-1\right)},\mu_{3,2}^{\left(t-1\right)},\ldots ,\mu_{3,K}^{\left(t-1\right)}\right],$$

where $\Delta R_{S}=R_{S}^{\left(t\right)}-R_{S}^{\left(t-1\right)}$. $\mu_{1}^{\left(t\right)},\mu_{2}^{\left(0\right)},\mu_{3,1}^{\left(0\right)},\mu_{3,2}^{\left(0\right)},\ldots ,\mu_{3,K}^{\left(0\right)}$ are initialized to zero. This set of inputs collectively provides the GRU with sufficient information to assess the current optimization state. The iteration index *t* indicates the stage of the optimization process, and the sum secrecy rate in the last iteration $R_{S}^{\left(t-1\right)}$ and its increment $\Delta R_{S}$ reflect the progress and convergence trend of the objective function. Together, they guide the GRU to adaptively output an appropriate step size for the subsequent iteration.

At each iteration, the GRU processes the input sequence and the hidden state from the previous iteration. The hidden state serves as a memory mechanism, allowing the network to retain information about the convergence history and temporal dynamics of the optimization process. The fully connected (FC layer) is employed to map the high-dimensional hidden state features to the specific step size values required for the optimization algorithm. This FC layer transforms the hidden state into the desired output dimension. Thus the output set of GRU is defined as

(29) $$output^{\left(t\right)}=\left[\mu_{1}^{\left(t\right)},\mu_{2}^{\left(t\right)},\mu_{3,1}^{\left(t\right)},\mu_{3,2}^{\left(t\right)},\ldots ,\mu_{3,K}^{\left(t\right)}\right].$$

Then the update of the optimization variables can be performed based on (12)–(27) in Section 3.1. To ensure stable training, we fix the iteration of the network to a predefined large value *T* during the training stage as shown in Figure 2. This value is chosen to cover the range of iterations that may be required during deployment, thereby allowing the network to sufficiently learn the optimization trajectory. During the deployment stage, the network can determine the required number of iterations via a convergence criterion, enabling efficient and adaptive optimization. We employ an unsupervised learning approach; therefore, the loss function is defined as

(30) $$L=\frac{1}{T}\sum_{t=1}^{T}R_{S}^{\left(t\right)}.$$

Finally, the GRU parameters are updated by the chosen optimizer.

During the deployment stage, when the convergence condition $\Delta R_{S}\leq \epsilon$ is satisfied, the iteration ends, thereby achieving dynamic iteration adjustment as shown in Figure 3. The training and deployment procedure of the proposed method are summarized in Algorithm 1.
**Algorithm 1** Training and deployment procedure of the proposed GRU-aided deep unfold network  1:  **Input:** Channel realizations and CSI error uncertainty bounds.  2:  *Training Procedure:*  3:  **Initialization:** Randomly initialize the parameters in GRU.  4:  **for** e=1 to E=1 **do**  5:        Randomly initialize $\theta^{\left(0\right)},\Delta h_{re,k}$. $W^{\left(0\right)}$ is initialized according to (11).  6:        Step sizes $\mu_{1}^{\left(0\right)},\mu_{2}^{\left(0\right)},\mu_{3,1}^{\left(0\right)},\mu_{3,2}^{\left(0\right)},\ldots ,\mu_{3,K}^{\left(0\right)}$ are initialized to 0.  7:        **for** t=1 to *T* **do**  8:              Generate step sizes via (28) and (29).  9:              Update $\Delta h_{re,k}$ via (12) to (15);10:              Update W via (16) to (21);11:              Update θ via (22) to (27);12:        **end for**13:        Compute loss function L via (30) and update GRU parameters.14:  **end for**15:  *Deployment Procedure:*16:  **Initialization:** Randomly initialize $\theta^{\left(0\right)},\Delta h_{re,k}$. $W^{\left(0\right)}$ is initialized according to (11).17:  Step sizes $\mu_{1}^{\left(0\right)},\mu_{2}^{\left(0\right)},\mu_{3,1}^{\left(0\right)},\mu_{3,2}^{\left(0\right)},\ldots ,\mu_{3,K}^{\left(0\right)}$ are initialized to 0.18:  **for** t=1 to *T* **do**19:        Generate step sizes via (28) and (29).20:        Update $\Delta h_{re,k}$ via (12) to (15);21:        Update W via (16) to (21);22:        Update θ via (22) to (27);23:        **if** $\Delta R_{S}\leq \epsilon$ **then** break;24:        **end if**25:  **end for**26:  **Output:** W,θ.

### 3.3. Computational Complexity Analysis

The complexity of the proposed deep unfold network is analyzed as follows. The complexity comes from the following parts. First, computing the gradient $\nabla_{\Delta h_{re,k}}R_{S}$ requires $O(KN^{2})$. Next, the complexity of obtaining the worst case CSI for all *K* Eves is $O(K^{2}N^{2})$. The complexity of updating W is $O(K^{3}M)$, while the complexity of updating θ is $O(K^{2}N^{2})$. Additionally, generating adaptive step sizes via the GRU and FC layer has a constant complexity denoted as O(c), where *c* depends only on the structure of the GRU and FC layer. In summary, for each iteration the total computational complexity amounts to $O(K^{3}M+K^{2}N^{2}+c)$.

## 4. Numerical Simulations and Analysis

In this section, we present numerical simulations to validate the effectiveness of the proposed model-driven deep learning approach. Some parameters are defined as follows. The path loss $C_{0}$ at the reference distance $D_{0}=1$ m is set to −30 dB, the path loss factor α=2.2 and the Rician factor κ=10. We assume that K=4. The locations of the nodes in the system are defined as follows. Alice is located at (0 m, 0 m, and 10 m), and RIS is located at (160 m, 20 m, and 5 m). Legitimate users are located in a circle centered at (200 m, 0 m, and 0 m) within a radius of 10 m. The eavesdroppers are located randomly in a circle centered at (180 m and 0 m) within a radius of 10 m. The weighted coefficient is set to $\psi_{k}=1/K$. The noise power at each receiver is set to $\sigma_{b,k}^{2}=\sigma_{e,k}^{2}=-80\;\mathrm{dBm}$. We define the uncertainty level as $\epsilon_{k}=\rho_{k}/\left|\right|h_{re,k}\left|\right|$ and $\epsilon_{k}$ is set to 0.05. The batch size B=10. The parameters of the GRU and FC layer are shown in Table 1. The dataset consists of 1000 channel realizations. We choose the Adam optimizer [41] to update the GRU parameters. The simulations are implemented in Python 3.8 and deep learning framework Pytorch 1.8 on a PC with Intel(R) Core(TM) i9-10980XE CPU @ 3.00GHz, 256 GB RAM and NVIDIA GeForce RTX 3090. To verify the effectiveness of the proposed GRU-aided deep unfold network, we choose the following methods as baselines:

- Non-robust AO [14]: Non-robust AO directly uses the estimated CSI of eavesdroppers to design the secure beamforming without considering CSI error. The precoder matrix is optimized through successive convex approximation (SCA) and the RIS phase shift matrix is optimized through alternating direction method of multiplier (ADMM).
- Deep unfold network with fixed number of layers: In this method, the algorithm in Section 3.1 is directly unfolded into trainable layers.
- No unfold: In this method, the step sizes are all initialized to 0.1 and not trained.
- WMMSE [42]: In this method, the precoder matrix is designed by weighted minimum mean square error (WMMSE) algorithm.

**Figure 4:**
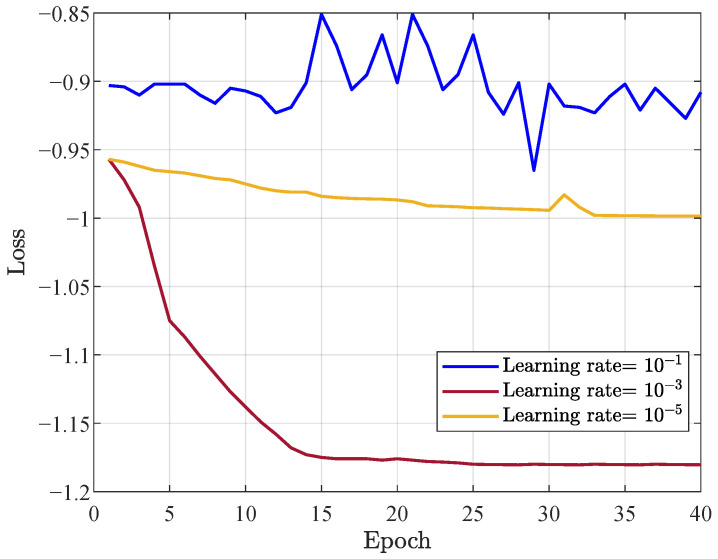
Convergence of the loss function.

In Figure 4, the convergence of the loss function at the training stage is presented. The learning rate exhibits a significant influence on the convergence behavior of the loss function. When the learning rate is set to $10^{-1}$, the update of the network parameters is large, leading to significant oscillation and non-convergence phenomena in the loss function. Conversely, a learning rate of $10^{-5}$ results in a small update step, which in turn leads to slow convergence. In contrast, with a learning rate of $10^{-3}$ the loss function decreases steadily and achieves effective convergence. Therefore, to balance convergence stability with training efficiency, a learning rate of $10^{-3}$ is adopted.

**Figure 5:**
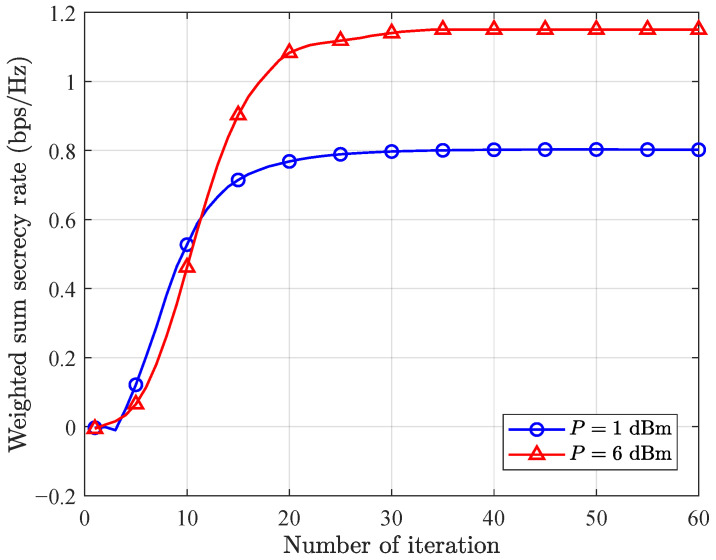
Weighted sum secrecy rate versus iterations.

In Figure 5, we present the convergence behavior of the proposed GRU-aided deep unfold network. It is observed that when P=1 dBm, the proposed method requires 30 iterations to converge, and when P=6 dBm, the proposed method requires 35 iterations to converge. As illustrated in the figure, the proposed algorithm demonstrates stable and effective convergence behavior. The reason is that we view the step size generation at each iteration as a sequence generation task, leveraging the inherent capability of GRU in processing sequential data to dynamically generate appropriate step sizes. Specifically, the GRU adaptively adjusts the step size based on the current iteration index and the trend of the objective function variation, thereby achieving efficient convergence. It is also observed that different *P* requires different iterations to converge, further verifying the importance of adaptive iteration adjustment in deep unfold networks to achieve satisfied performance.

**Figure 6:**
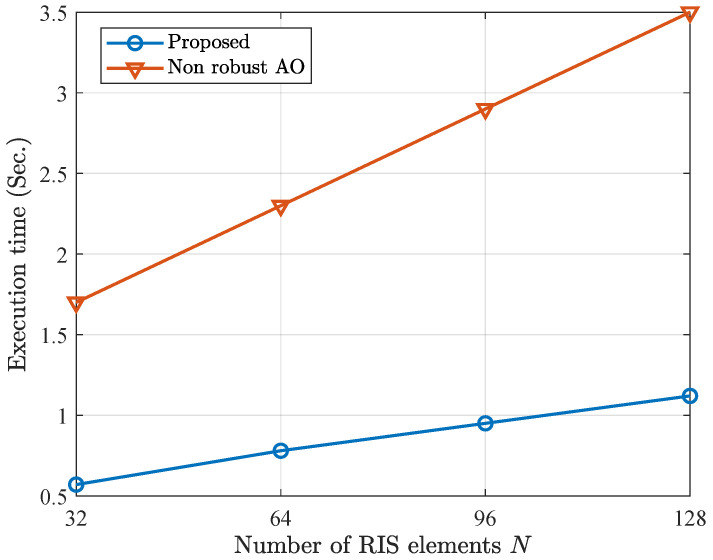
Execution time versus number of RIS elements.

In Figure 6, we demonstrate the execution time versus number of RIS elements. It is observed that the proposed method has a significantly lower execution time than that of the non-robust AO. This demonstrates the advantage of our proposed method in terms of execution time. The proposed method utilizes a GRU to generate the step size for each iteration. During the deployment stage, only the forward propagation of the network is required, leading to low computational overhead. In addition, the number of parameters of GRU remains independent of the number of RIS elements *N*. Consequently, the overall computational complexity of the algorithm does not increase significantly with the problem scale, thereby maintaining relatively low computational complexity.

**Figure 7:**
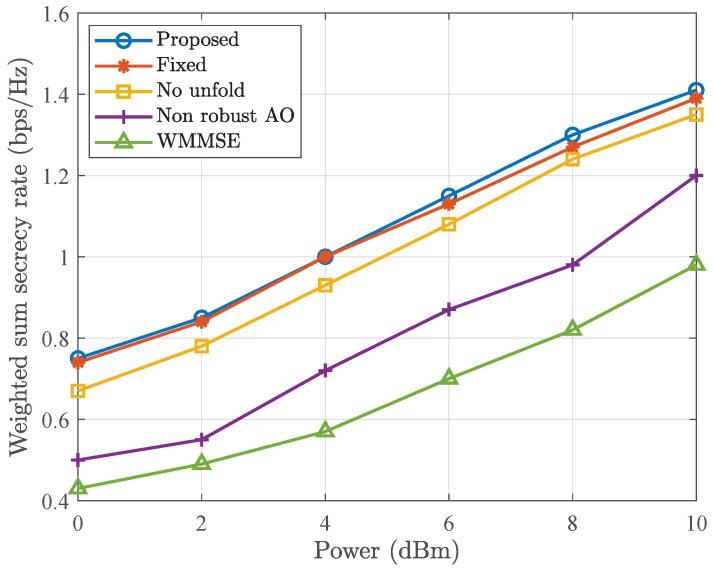
Weighted sum secrecy rate versus transmit power *P*.

In Figure 7, we demonstrate the weighted sum security rate versus transmit power *P*. It can be observed that as *P* increases, the weighted sum secrecy rate increases accordingly, and the proposed robust beamforming method achieves a higher weighted sum secrecy rate than non-robust AO. Specifically, when P=6 dBm, the weighted sum secrecy rate of the GRU-aided deep unfold network, traditional deep unfold network, non-robust AO and WMMSE are 1.15 bps/Hz, 1.13 bps/Hz, 1.08 0.87 bps/Hz and 0.71 bps/Hz, respectively. The results indicate that compared to non-robust AO, the proposed robust method improves performance by approximately 0.28 bps/Hz. This improvement is attributed to the fact that the proposed robust method considers the channel uncertainty and performs robustness optimization. The robust design ensures that the system can still maintain robust performance within the CSI uncertainty range. In contrast, the weighted sum secrecy rate of the no unfold method is slightly lower due to the untrained step size. And the non-robust AO method only designs beamforming based on the estimated CSI, without considering the CSI error. Therefore, it is more sensitive to CSI imperfection. The WMMSE method only optimize the precoder matrix with random RIS phase shift and it has the lowest sum secrecy rate. In addition, it can be seen that when P>6 dBm, the proposed GRU-aided deep unfold network can achieve higher weighted sum secrecy rate than traditional deep unfolding networks. The reason is that the GRU-aided method applies adaptive layer adjustment design, which overcomes the performance limitations caused by fixed layer design.

**Figure 8:**
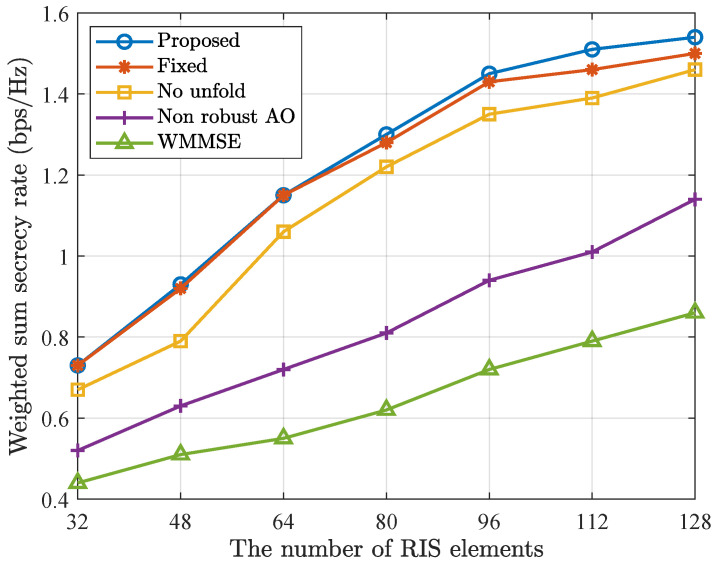
Weighted sum secrecy rate versus number of RIS elements *N*.

The impact of the number of RIS elements *N* on weighted sum secrecy rate is examined in Figure 8. As *N* increases, the beamforming gain provided by the RIS is enhanced, which in turn improves the weighted sum secrecy rate of all methods increases accordingly. In addition, as *N* increases, the complexity of the optimization grows. It requires more iterations to converge. However, existing deep unfolding networks, due to the fixed number of layers, lack the flexibility to adapt to this increased iteration demand. Consequently, their performance is limited when solving high-dimensional problems. Notably, the proposed method consistently outperforms other baselines, demonstrating its superior performance.

**Figure 9:**
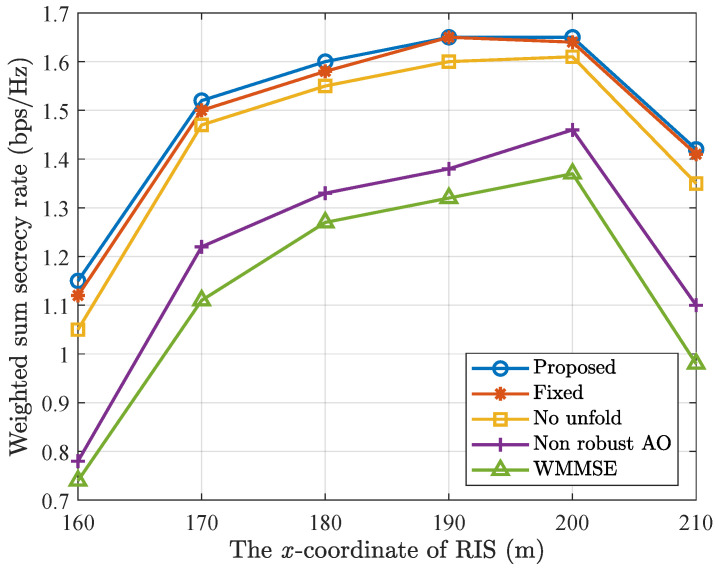
Weighted sum secrecy rate versus the *x*-coordinate of RIS location.

In Figure 9, we investigate the impact of the different RIS locations on the weighted sum secrecy rate. We slowly move RIS from (160 m and 20 m) to (210 m and 20 m). It is observed that when x ∈ [160 m, 200 m], RIS approaches legitimate users and the weighted sum secrecy rate increases, and when x ∈ [200 m, 210 m], RIS gradually moves away from the legitimate users, and the weighted sum secrecy rate decreases. This phenomenon can be attributed to the fact that when x = 200 m, the distance between RIS and legitimate users is the nearest, the beamforming gain is the highest. Therefore the effect to suppress the leakage to the eavesdropper is the least.

**Figure 10:**
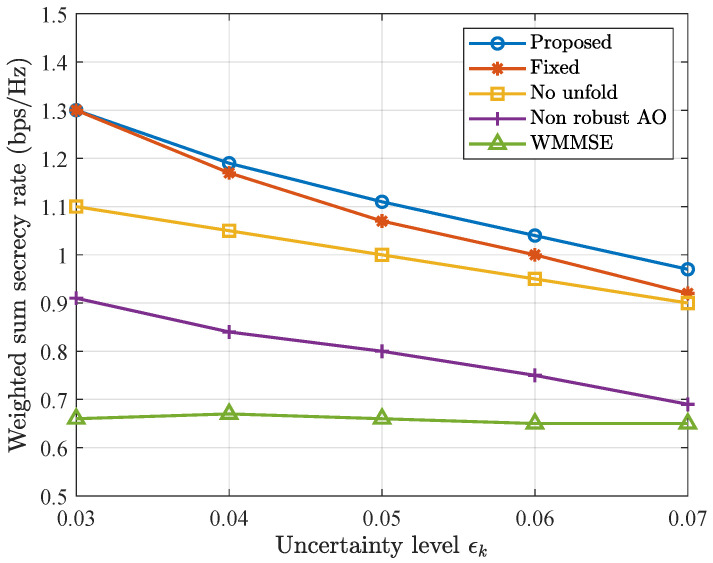
Weighted sum secrecy rate versus uncertainty level.

In Figure 10, the weighted sum secrecy rate versus channel uncertainty level is illustrated. It is observed that as the uncertainty level increases, the performance of all methods decreases and the proposed method always outperforms other baselines. The WMMSE algorithm optimizes the precoder matrix without considering the eavesdropper; therefore, its performance is the lowest and not sensitive to eavesdropper’s channel uncertainty level.

**Figure 11:**
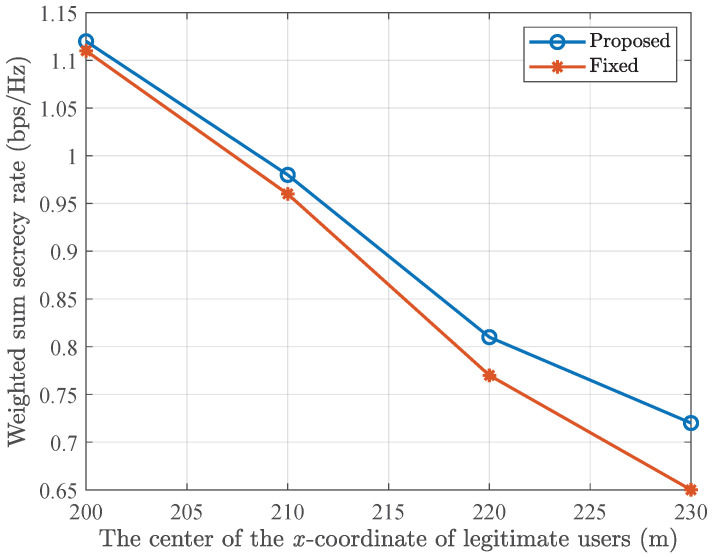
Weighted sum secrecy rate versus the location of the legitimate users.

In Figure 11, we illustrates the weighted sum secrecy rate versus the location of legitimate users. It is observed that as the legitimate users move farther away gradually, the weighted sum secrecy rate decreases. This is primarily due to the increased distance between the RIS and the users, which leads to greater path loss and thus reduces the weighted sum secrecy rate. Additionally, it is shown that as the performance gap between the proposed GRU-aided and traditional deep unfold network increases. This occurs because as the legitimate users move away, the optimization problem requires more iterations to converge. The proposed method leverages GRU to adjust the iteration, while the traditional deep unfold network fails to meet this increased iteration demand, resulting in performance loss. This further validates the effectiveness of the proposed method for robust and secure beamforming.

## 5. Conclusions

In this paper, we investigated the physical layer security in RIS-aided MU-MISO system in the presence of multiple eavesdroppers. In practice, imperfect CSI presents a critical challenge for RIS-aided physical layer security design. To address this problem, we proposed a robust model-driven deep unfold network. First, we introduced the gradient descent–ascent algorithm to solve the original optimization problem. Building upon this iterative structure, we further introduced an GRU-aided deep unfolding network. This design effectively integrates the sequential learning capability of GRU with the model-based iterative algorithm. The proposed deep unfolding network utilizes the GRU to adaptively generate the gradient update step size for each iteration. The proposed method introduces a flexible iteration adjustment mechanism, enabling the network to dynamically determine the required number of iterations based on convergence conditions. The simulation results demonstrate that compared to existing non-robust optimization algorithms and the traditional deep unfold network with a fixed number of iterations, the proposed method exhibits robustness against imperfect CSI and achieves a higher weighted sum secrecy rate.

## Figures and Tables

**Table 1:** The parameters of the GRU and FC layer.

Parameters	Values
Hidden Size of GRU	16
Input Size of FC Layer	16
Neurons in the Hidden Layer of FC Layer	32
Output Size of FC Layer	2+K
Activation Function	ReLU

## Data Availability

All data generated or analyzed during this study are included in this article.

## References

[1] Wang C.-X., You X., Gao X., Zhu X., Li Z., Zhang C. (2023). On the Road to 6G: Visions, Requirements, Key Technologies, and Testbeds. IEEE Commun. Surv. Tutor..

[2] Butun I., Österberg P., Song H. (2020). Security of the Internet of Things: Vulnerabilities, Attacks, and Countermeasures. IEEE Commun. Surv. Tutor..

[3] Hong Y.W.P., Lan P.C., Kuo C.C.J. (2013). Enhancing Physical-Layer Secrecy in Multi-antenna Wireless Systems: An Overview of Signal Processing Approaches. IEEE Signal Process. Mag..

[4] Wu Q., Zhang R. (2020). Towards Smart and Reconfigurable Environment: Intelligent Reflecting Surface Aided Wireless Network. IEEE Commun. Mag..

[5] Zhao K., Song Z., Li Y., Li X., Liu L., Wang B., Li C. (2026). RIS-Aided Communication Network Deployment Strategy Based on BP Model: From Single-Hop to Multi-Hop. IEEE Trans. Green Comm. Netw..

[6] Zhu Z., Ning M., Sun G., Chu Z., Liu P., Ai B., Lee I. (2025). The Interplay of DMA and RIS for Near-Field Integrated Sensing and Symbiotic Radio Systems. IEEE Internet Things J..

[7] Zhu Z., Guo K., Chu Z., Mi D., Mu J., Muhaidat S., Wong K. (2025). Unlocking Integrated Wireless Powered Sensing and Communication Networks Using Reconfigurable Intelligent Surface. IEEE Trans. Wirel. Commun..

[8] Zhu Z., Gong M., Zhang M., Wu Q., Sun G., Chu Z., Li X., Lee I. (2025). Reinforcement Learning Based Resource Allocation in IRS Assisted SWIPT Systems. IEEE Trans. Veh. Technol..

[9] Guo M., Lin Z., Ma R., An K., Li D., Al-Dhahir N., Wang J. (2024). Inspiring Physical Layer Security with RIS: Principles, Applications, and Challenges. IEEE Open J. Commun. Soc..

[10] Cui M., Zhang G., Zhang R. (2019). Secure Wireless Communication via Intelligent Reflecting Surface. IEEE Wireless Commun. Lett..

[11] Feng K., Li X., Han Y., Jin S., Chen Y. (2021). Physical Layer Security Enhancement Exploiting Intelligent Reflecting Surface. IEEE Commun. Lett..

[12] Shen H., Xu W., Gong S., He Z., Zhao C. (2019). Secrecy Rate Maximization for Intelligent Reflecting Surface Assisted Multi-Antenna Communications. IEEE Commun. Lett..

[13] Cheng Z., Li N., Zhu J., She X., Ouyang C., Chen P. (2023). RIS-Assisted Secure Communications: Low-Complexity Beamforming Design. IEEE Wireless Commun. Lett..

[14] Niu H., Chu Z., Zhou F., Zhu Z., Zhang M., Wong K.K. (2021). Weighted Sum Secrecy Rate Maximization Using Intelligent Reflecting Surface. IEEE Trans. Commun..

[15] Kilpi-Chen X., Chang Z., Hämäläinen T. (2025). Secure Transmission for IRS-on-UAV-Assisted Wireless Networks. IEEE Trans. Commun..

[16] Kamal M.M., Abideen S.Z.U., Shah S.S., Sehito N., Khan S., Virdee B.S., Alibakhshikenari M., Livreri P. (2025). Secure Satellite Downlink with Hybrid RIS and AI-Based Optimization. IEEE Access.

[17] Jiang C., Zhang C., Huang C., Ge J., Niyato D., Yuen C. (2025). RIS-Assisted ISAC Systems for Robust Secure Transmission with Imperfect Sense Estimation. IEEE Trans. Wireless Commun..

[18] Yu X., Xu D., Sun Y., Ng D.W.K., Schober R. (2020). Robust and Secure Wireless Communications via Intelligent Reflecting Surfaces. IEEE J. Sel. Areas Commun..

[19] Zhai Z., Lei W., Lei H., Tang H. (2024). Robust Design of the Security Scheme in IRS-Assisted MISO Systems with Imperfect Eavesdropping CSI. IEEE Trans. Veh. Technol..

[20] Bai J., Wang H.M., Liu P. (2022). Robust IRS-Aided Secrecy Transmission With Location Optimization. IEEE Trans. Commun..

[21] Qin Z., Ye H., Li G.Y., Juang B.H.F. (2019). Deep Learning in Physical Layer Communications. IEEE Wireless Commun..

[22] Guo Y., Zhang J., Hong Y.W.P., Tomasin S. (2026). Model-Driven Learning-Based Physical Layer Authentication for Mobile Wi-Fi Devices. IEEE Trans. Inf. Forensics Secur..

[23] Jin W., Zhang J., Wen C.K., Jin S., Zheng F.C. (2025). Joint Beamforming in RIS-Assisted Multi-User Transmission Design: A Model-Driven Deep Reinforcement Learning Framework. IEEE Trans. Commun..

[24] Wang J., Tang W., Han Y., Jin S., Li X., Wen C.K., Cheng Q., Cui T.J. (2021). Interplay Between RIS and AI in Wireless Communications: Fundamentals, Architectures, Applications, and Open Research Problems. IEEE J. Sel. Areas Commun..

[25] Jiang P., Wen C.K., Yi X., Li X., Jin S., Zhang J. (2024). Semantic Communications Using Foundation Models: Design Approaches and Open Issues. IEEE Wireless Commun..

[26] Zhu Z., Wang H., Sun G., Li X., Shen Z., Liu Y., Zhang J. (2024). Coupled Phase-Shift STAR-RIS for Secure MIMO Communication: A DRL-Based Beamforming Design. IEEE Wireless Commun..

[27] Xu W., Gan L., Huang C. (2022). A Robust Deep Learning-Based Beamforming Design for RIS-Assisted Multiuser MISO Communications With Practical Constraints. IEEE Trans. Cognit. Commun. Netw..

[28] Deka S., Deka K., Nguyen N.T., Sharma S., Bhatia V., Rajatheva N. (2026). Comprehensive Review of Deep Unfolding Techniques for Next-Generation Wireless Communication Systems. IEEE Internet Things J..

[29] Ding C., Jin W., Li X., Matthaiou M., Yi X., Jin S. (2025). Multi-Group Multicasting Using Reconfigurable Intelligent Surfaces: A Deep Learning Approach. IEEE Trans. Wireless Commun..

[30] Jin W., Zhang J., Wen C.K., Jin S. (2023). Model-Driven Deep Learning for Hybrid Precoding in Millimeter Wave MU-MIMO System. IEEE Trans. Commun..

[31] Chowdhury A., Verma G., Swami A., Segarra S. (2024). Deep Graph Unfolding for Beamforming in MU-MIMO Interference Networks. IEEE Trans. Wireless Commun..

[32] Wang X., Zhu F., Huang C., Alhammadi A., Bader F., Zhang Z., Yuen C., Debbah M. (2024). Robust Beamforming with Gradient-Based Liquid Neural Network. IEEE Wireless Commun. Lett..

[33] Lavi O., Shlezinger N. (2023). Learn to Rapidly and Robustly Optimize Hybrid Precoding. IEEE Trans. Commun..

[34] Liu W., Xu H., He X., Ye Y., Zhou A. (2024). Bi-Level Deep Unfolding Based Robust Beamforming Design for IRS-Assisted ISAC System. IEEE Access.

[35] Abdallah A., Celik A., Mansour M.M., Eltawil A.M. (2023). RIS-Aided mmWave MIMO Channel Estimation Using Deep Learning and Compressive Sensing. IEEE Trans. Wireless Commun..

[36] Wang P., Fang J., Duan H., Li H. (2020). Compressed Channel Estimation for Intelligent Reflecting Surface-Assisted Millimeter Wave Systems. IEEE Signal Process. Lett..

[37] Mukherjee A., Swindlehurst A.L. Detecting passive eavesdroppers in the MIMO wiretap channel. Proceedings of the IEEE International Conference on Acoustics, Speech and Signal Processing (ICASSP).

[38] Liu Z., Zhu B., Xie Y., Ma K., Guan X. (2024). UAV-Aided Secure Communication with Imperfect Eavesdropper Location: Robust Design for Jamming Power and Trajectory. IEEE Trans. Veh. Technol..

[39] Razaviyayn M., Huang T., Lu S., Nouiehed M., Sanjabi M., Hong M. (2020). Nonconvex Min-Max Optimization: Applications, Challenges, and Recent Theoretical Advances. IEEE Signal Process. Mag..

[40] Tang J., Lv Z., Xiao J., Wu J., Shim B. (2026). Weighted Sum-Rate Maximization by Joint Antenna Grouping and Movable RIS Deployment. IEEE Trans. Wireless Commun..

[41] Kingma D.P., Ba J. (2014). Adam: A method for stochastic optimization. arXiv.

[42] Shi Q., Razaviyayn M., Luo Z.Q., He C. (2011). An Iteratively Weighted MMSE Approach to Distributed Sum-Utility Maximization for a MIMO Interfering Broadcast Channel. IEEE Trans. Signal Process..

